# Potential Immunomodulatory Activity of a Selected Strain *Bifidobacterium bifidum* H3-R2 as Evidenced *in vitro* and in Immunosuppressed Mice

**DOI:** 10.3389/fmicb.2020.02089

**Published:** 2020-09-01

**Authors:** Jiacui Shang, Feng Wan, Le Zhao, Xiangchen Meng, Bailiang Li

**Affiliations:** ^1^Key Laboratory of Dairy Science, Ministry of Education, Northeast Agricultural University, Harbin, China; ^2^School of Food Science, Northeast Agricultural University, Harbin, China

**Keywords:** *B. bifidum* H3-R2, cytoxan, immunosuppress, immune cell, immunoregulation

## Abstract

The microbiota is directly involved in the development and modulation of the intestinal immune system. In particular, members of the genus *Bifidobacterium* play a primary role in immune regulation. In the present study, *Bifidobacterium bifidum* H3-R2 was screened from 15 bifidobacterium strains by *in vitro* experiment, showing a positive tolerance to digestive tract conditions, adhesion ability to intestinal epithelial cells and a regulatory effect on immune cell activity. Immunostimulatory activity of *B. bifidum* H3-R2 was also elucidated *in vivo* in cytoxan (CTX)-treated mice. The results showed that the administration of *B. bifidum* H3-R2 ameliorated the CTX-induced bodyweight loss and imbalanced expression of inflammatory cytokines, enhanced the production of secretory immunoglobulin A (SIgA), and promoted splenic lymphocyte proliferation, natural killer (NK) cell activity and phagocytosis of macrophages in immunosuppressed mice. In addition, *B. bifidum* H3-R2 restored injured intestinal mucosal, and increased the villus length and crypt depth in CTX-treated mice. The results could be helpful for understanding the functions of *B. bifidum* H3-R2, supporting its potential as a novel probiotic for immunoregulation.

## Introduction

The gut microbiota ecosystem has a profound influence on host health (Cox et al., 2014; Dong and Gupta, 2019), and is directly involved in the development and modulation of the immune system by enhancing host defense mechanisms such as promoting mucosal barrier function and enhancing immune responses (Kamada et al., 2013; Matamoros et al., 2013; Feng et al., 2018). However, several endogenous and environmental factors can have impacts on both the composition and functionality of the gut microbiota throughout life (Candela et al., 2015; Sonnenburg and Sonnenburg, 2019).

Bifidobacteria are a dominant group of the gut microbiota and play an important role in promoting a favorable gut ecosystem and possess immunoregulatory effects in animals and humans (Vuyst and Leroy, 2011; Hill et al., 2017). Some species of *Bifidobacterium* have been found to beneficially coexist with other members of the commensal microbiota (Turroni et al., 2016; Wong et al., 2019) and affect the immune system (Philippe et al., 2011; Tsai et al., 2012; Routy et al., 2018). This is based on the interaction of a specific bifidobacteria molecule, a microbe-associated molecular pattern (MAMP), with a pattern recognition receptor (PRR) present on the membrane of epithelial/immune cells, which mostly configures the cellular structure of the intestinal mucosa (Sutterwala and Flavell, 2009; Ruiz et al., 2017). Not all bifidobacteria have equal activities in promoting immunological function (Pozo-Rubio et al., 2011; Mazen et al., 2018).

Cytoxan (CTX) is a potent immunosuppressive agent with cytotoxic and immunosuppressive activities. The inactive CTX must first undergo metabolic activation, catalyzed by the hepatic cytochrome P450 to 4-hydroxy-cyclophosphamide and then to phosphoramide mustard and acrolein. The mustard component produces a cytotoxic effect by preventing cell replication, while acrolein is linked with toxic side effects (Sun and Peng, 2008; Yu et al., 2014), which were characterized by damaging the intestinal barrier, inducing immune cell damage and altering the composition of the gut microbiota (Ding et al., 2019; Sun et al., 2019). At present, the immunoregulatory effect of bifidobacteria is mainly focused on studies in normal mice and inflammation of mice, while there are few studies on immunosuppressed mice caused by CTX. Therefore, CTX injection, a classical modeling method, was used in this study to evaluate the immunoregulatory effect of bifidobacteria on immunosuppressed mice.

To make a rational choice from available bifidobacterial strains, we first performed an *in vitro* screen of potential probiotics by estimating survival and persistence in the simulated digestive tract and regulation of immune cell activity. We also further investigated the strain-specific immunostimulatory effects in CTX-induced immunosuppressed mice. The purpose of this work was to evaluate the characteristics of a new probiotic strain and provide a probiotic strain with immunoregulatory functions for development in food and pharmaceutical.

## Materials and Methods

### Preparation of Bacterial Strains

*Bifidobacterium longum* subsp. *longum* H3-R1, H9-5, H10-1, H16-2, H20-14, and H27-10, *Bifidobacterium animalis* subsp. *lactis* H15-2, H27-9, and H34-21, *Bifidobacterium bifidum* H3-R2 and H10-5, *B. longum* subsp. *infantis* H5-21 and H11, *Bifidobacterium breve* H34-14 and *Bifidobacterium pseudocatenulatum* H17-2 were isolated from exclusively breast-fed infants and stored at the Key Laboratory of Dairy Science (KLDS), Ministry of Education, China. *B. animalis* subsp. *lactis* BB12 (ATCC 27536) was used as a reference strain for screening bifidobacteria with immunostimulatory activity. All strains were anaerobically incubated in De Man, Rogosa and Sharpe (MRS) (Oxoid, United Kingdom) medium supplemented with 0.05% L-cysteine hydrochloride (mMRS) at 37°C for 18 h and were sub-cultured twice prior to the experiment. Bacteria were harvested by centrifugation (8000 rpm for 5 min at 4°C) and the cells were washed with saline buffer. The cell pellets were resuspended to the dosages as specified in the relevant sections for the *in vitro* and *in vivo* work.

### Assay of Tolerance to Simulated Digestive Tract Conditions

The simulated digestive tract model consisted of three processes, including the mouth, stomach and small intestine. Artificial saliva was prepared by suspending 3 g/L α-amylase (Sigma, United States) in sterile solution (6.2 g/L NaCl, 2.2 g/L KCl, 0.22 g/L CaCl 2, and 1.2 g/L NaHCO_3_), then the pH was adjusted to 6.9 (Barmpalia-Davis et al., 2009). Simulated gastric fluid was prepared by dissolving 3.0 g/L pepsin (Sigma, United States) from porcine stomach mucosa in sterile solution (3 g/L NaCl, 1.1 g/L KCl, 0.15 g/L CaCl_2_, and 0.6 g/L NaHCO_3_), then the pH was adjusted to 2 with HCl. Artificial small intestinal fluid was prepared by dissolving 3 g/L oxgall (Sigma, United States), 1 g/L pancreatin (Sigma, United States), and 0.1 g/L lipase (Sigma, United States) in sterile solution (5 g/L NaCl, 0.6 g/L KCl, 0.3 g/L CaCl_2_, and 0.6 g/L NaHCO_3_). The solution was filtered through a 0.22-μm filter prior to use (Khalf et al., 2010). Firstly, bacterial cells (5 mL, 1 × 10^9^ CFUs/mL) were suspended in 5 mL saliva (pH 6.9) for 5 min. Then bacterial cells were centrifuged and resuspended in 10 mL gastric fluid and incubated for 2 h. Finally, the bacteria were centrifuged and resuspended in 10 mL intestinal fluid and incubated for 2 h. The entire digestion procedure was performed at 37°C, with stirring at 50 rpm to simulate peristaltic contraction (Weiss and Jespersen, 2010). The bacteria suspensions were diluted 10-fold and plated on mMRS agar plates. The number of colonies was counted following 36 h of incubation at 37°C under anaerobic conditions. N_1_ represents the total count of strains after treatment. N_0_ represents the total count of strains before treatment.

$$\mathrm{Survival}\mathrm{rate}\left(\%\right)=\frac{\mathrm{Log}\,N_{1}}{\mathrm{Log}\,N_{0}}\times 100$$

### Assay of Adhesion to Intestinal Epithelial Cell

In order to examine cell adhesion of bifidobacteria and reference strain to human intestinal cells, the human colon adenocarcinoma (Caco-2) cell line was obtained from China Cell Bank. Caco-2 cells were cultured in Dulbecco’s Modified Eagle’s Medium (DMEM) (Hyclone, United States) supplemented with 10% fetal bovine serum (FBS) (Wisent, Canada) and 1% penicillin-streptomycin (Ameresco, United States) at 37°C, 5% CO_2_. For the adherence assays, cells were seeded at a concentration of 2 × 10^5^ cells per well in 12-well culture plates and incubated until the confluent. Bacteria were seeded at a concentration of 10^8^ colony-forming units (CFUs) per well. After co-culture for 2 h, each well was washed four times with PBS. The cells were treated with 250 μL trypsin (Sigma, United States) and incubated at 37°C for 3 min. Then each well was added 250 μL DMEM supplemented with 10% FBS to terminate the activity of trypsin. The bound bacteria were subjected to 10-fold serial dilution and plated onto mMRS agar plates. The number of colonies was counted following 36 h of incubation at 37°C under anaerobic conditions. C_1_ represents the total count of adhesive strains after treatment. C_0_ represents the total count of strains before treatment.

$$\mathrm{Adhesion}\mathrm{rate}\left(\%\right)=\frac{C_{1}}{C_{0}}\times 100$$

### Assay of Proliferation of Splenic Lymphocyte *in vitro*

Assay was performed with modifications to a previously published method (Peng et al., 2014). The spleen was aseptically removed from the mice and placed into 3 mL Hank’s solution (Ameresco, United States) on a small sterile dish, ground, and filtered through nylon mesh. The cells were collected by centrifugation (1000 rpm, 10 min). Tris−NH_4_Cl lysis buffer (Solarbio, China) kept at 37°C was added to the mixture for 5 min to lyse the red blood cells (RBC) thoroughly. The splenocytes were adjusted to 2 × 10^6^ cells/mL in Roswell Park Memorial Institute 1640 (RPMI-1640) (Hyclone, United States) medium supplemented with 10% FBS and seeded into a 96-well plate at density of 1 × 10^6^ cells/well. Then 20 μL Concanavalin A (5 μg/mL) (Sigma, United States) was added to each well. Splenocytes were treated in presence of bifidobacteria at the ratios of 100:1, 10:1, and 1:1 (bacteria/cells), respectively for 36 h. The positive group was not treated with bifidobacteria. The negative group was not treated with splenocytes. The Cell Counting Kit-8 (CCK-8) (Sigma, United States) solution were added to each well and cultured for 2 h. The absorbance was measured at 450 nm using a microplate reader (Beckman, United States). OD_1_ represents the optical density value of bifidobacteria-treated group, OD2 represents the optical density value of negative group, and OD_3_ represents the optical density value of positive group. The proliferation index (PI) of splenic lymphocyte was calculated according to the following formula:

$$\text{PI}=\,\frac{{\text{OD}}_{1}-{\text{OD}}_{2}}{{\text{OD}}_{3}}$$

### Assay of Phagocytosis of Peritoneal Macrophages *in vitro*

Mice were anesthetized and sacrificed. A sterile syringe was used to slowly inject 3 mL of RPMI-1640 (Hyclone, United States) medium into the peritoneal cavity, which was then gently pressed and rubbed for 2 min (Lim and Kim, 2017). Peritoneal fluid was extracted into centrifuge tube. Then the peritoneal cavity was washed with RPMI-1640 medium and the peritoneal fluid was centrifuged at 1000 rpm for 10 min. The cells were adjusted to 2 × 10^6^ cells/mL in RPMI-1640 medium supplemented with 10% FBS. 1 ml cell suspension was seeded in petri dish and maintained in a humidified incubator at 37°C with 5% CO_2_ for 3 h. Non-adhered cells were washed off and attached cells were used as macrophages. Macrophages were seeded at a density of 1 × 10^5^ cells/well in 96-well polystyrene plates and cultured for 6 h. Macrophages were treated in presence of bifidobacteria at the ratios of 100:1, 10:1, and 1:1 (bacteria/cells) for 24 h, respectively. The control group was not treated with bifidobacteria. One hundred microliters 0.075% neutral red (Biosharp, China) per well was added and incubated for 2 h. Culture medium was discarded and cells were washed with PBS 3 times to remove the neutral red that was not phagocytized by macrophage cells. Then, cell lysis buffer (Solarbio, China) was added and cultured for 4 h. The absorbance at 570 nm was measured using a microplate reader (Chen et al., 2010). The phagocytic capability of peritoneal macrophages was determined according to the following formula:

$$\mathrm{Phagocytic}\,\mathrm{capability}\,\left(\text{OD}\right)={\mathrm{OD}}_{\text{treatment}}\,{\text{OD}}_{\text{control}}$$

### Animals and Experimental Design

Specific Pathogen-Free (SPF) female BALB/c mice (7-week-old) were obtained from Beijing Vital River Laboratory Animal Technology Co., Ltd. BALB/c mice were housed in a controlled environment at an ambient temperature of 23 ± 2°C and a lighting regimen of 12 h light-dark cycles with free access to diet and drinking water. Mice with body weight of 15 ± 2.0 g were randomly divided into six groups (11 mice per group) after 1 week. The normal control group (NC) mice were gavaged with saline once a day during the experimental period. The CTX-induced model group (CM) mice received intraperitoneal injection of 80 mg/kg/d of CTX (Huayang, China) for three consecutive days and then were gavaged with saline for the next 28 days. The positive control group (PC) (immunosuppressed mice model) were gavaged with commercial strain BB12 at a dose of 10^8^ CFUs once a day (200 μl) for the next 28 days. The different doses of bifidobacterial intervention groups [high dose groups (BH), middle dose groups (BM) and low dose groups (BL)] were gavaged with *B. bifidum* H3-R2 at a dose of 10^10^, 10^8^, and 10^6^ CFUs respectively once daily at the same time for the next 28 days, and the control groups were treated with an equal volume of normal saline (200 μl) by oral gavage once daily at the same feeding frequency. After the experimental period, mice were sacrificed after anesthesia. Experimental protocol was approved by the Institutional Animal Care and Use Committee of the Northeast Agricultural University under the approved protocol number Specific pathogen free rodent management (SRM)-061.

### Assay of Immune Organs Index

Mice were weighed before being sacrificed. The thymus and spleen were immediately removed aseptically. The immune organ index was calculated according to the following formula:

$$\mathrm{Organ}\,\mathrm{index}\,\left(\text{mg}/\text{g}\right)\,=\frac{\mathrm{Spleen}\,\mathrm{or}\,\mathrm{thymus}\,\mathrm{weight}}{\mathrm{Body}\,\mathrm{weight}}$$

### Histopathological Examinations

The duodenum samples were washed with normal saline and immersed in 10% neutral-buffered formalin for 24 h at room temperature. The tissue sections were stained by hematoxylin and eosin (H&E) (Solarbio, China). The height of intestinal villi and depth of intestinal crypts were measured using image analysis software (Leica QWin, Germany).

### Assay of Proliferation of Splenic Lymphocyte

Splenocytes were prepared as above. The cells were adjusted to 2 × 10^6^ cells/mL in RPMI-1640 medium supplemented with 10% FBS and seeded into a 96-well plate at density of 4 × 10^5^ cells/well. Then 20 μL Concanavalin A (5 μg/mL) (Sigma, United States) was added to each well. After incubation for 48 h, the CCK-8 solution was added to each well and cultured for 2 h. The absorbance was measured at 450 nm using a microplate reader (Beckman, United States).

### Assay of Natural Killer (NK) Cell Activity

Natural killer (NK) cell activity was determined by CCK-8 assay (Yuan et al., 2010). Splenocytes were prepared as above. The concentration of YAC-1 lymphoma target cells (ATCC TIB-160) was adjusted to 1 × 10^5^ cells/ml with RPMI-1640 medium containing 10% FBS, and 100 ul per well YAC-1 target cells were added to 96-well plates. The splenocytes and YAC-1 cells were added with 10:1 ratio of effectors to target. The plates were then incubated at 37°C for 24 h. Next, 15 μL of CCK-8 solution was added to each well. Following 2 h of co-culture, the absorbance at 450 nm was measured using a microplate reader. OD_1_ represents the optical density value of test samples, OD_2_ represents the optical density value of effector cell control, and OD_3_ represents the optical density value of target cell control. The percentage of NK cell activity was determined by the following equation:

$$\mathrm{NK}\mathrm{cell}\mathrm{activity}\left(\%\right)=\left[1-\frac{{\text{OD}}_{1}-{\text{OD}}_{2}}{{\text{OD}}_{3}}\right]\times 100$$

### Assay of Peritoneal Macrophage Viability

Peritoneal macrophages were harvested by peritoneal lavage as above. The cells were seeded at a density of 2 × 10^5^ cells/well in 96-well polystyrene plates. The culture plates were maintained in a humidified incubator at 37°C with 5% CO_2_ for 26 h. The CCK-8 solution were added to the wells and incubated for 2 h. The absorbance was measured at 450 nm using a microplate reader (Beckman, United States) (Lim and Kim, 2017).

### Determination of Phagocytosis of Peritoneal Macrophage

Mice were intraperitoneally injected with 2% sheep red blood cells (SRBCs) (Bersee, China) to activate macrophages for 4 days before sacrifice. Peritoneal macrophages were collected by peritoneal lavage. The chicken red blood cells (CRBCs) (Bersée, China) were added to culture plates with macrophages at the ratio equal to 1:1 (v/v) and co-incubated for 30 min at 37°C. The plates were washed to remove non-engulfed CRBCs, and macrophages were fixed with methanol and stained with Giemsa (Biosharp, China). The cells were observed and counted using a microscope, and the percent of phagocytosis was calculated as follows (Cheng et al., 2010):

Phagocyticrate(%)=

(1) $$\frac{\mathrm{Number}\,\mathrm{of}\,\mathrm{macrophages}\,\mathrm{that}\,\mathrm{engulfed}\,\mathrm{CRBCs}}{100\,\mathrm{macrophages}}\times 100$$

### Assay of Carbon Clearance

The test of carbon clearance was carried out with some modifications from the described method (Huang and Ning, 2010). Macrophages were activated by SRBCs as described above. India ink (Amresco, United States) was injected into the tail vein of mice at a dose of 0.01 mL/g body weight. Blood was collected from the retro-orbital venous plexus at 2 min (t_1_) and 20 min (t_2_) time points after injection. Blood was mixed with 2 mL 0.1% Na_2_CO_3_ and absorbance was determined at 600 nm. OD_1_ and OD_2_ are the optical densities at 2 min (t_1_) and 20 min (t_2_), respectively. The mice were sacrificed and the livers and spleens were removed for weighing. The clearance index (K) and phagocytosis index (α) of carbon particles were calculated as follows: 

$$K\,=\,\frac{\mathrm{Log}\,{\mathrm{OD}}_{1}\,-\mathrm{Log}\,{\mathrm{OD}}_{2}}{{\text{t}}_{2}\,-t_{1}}$$

$$\alpha =\frac{\mathrm{Body}\,\mathrm{weight}}{\mathrm{Weights}\,\mathrm{of}\,\mathrm{spleen}\,\mathrm{and}\,\mathrm{liver}}\times \sqrt[{3}]{K}$$

### Determination of Cytokines in Serum

Blood samples were centrifuged at 3000 rpm for 20 min and the upper serum was collected. The levels of interleukin-10 (IL-10) and tumor necrosis factor-α (TNF-α) in serum were analyzed using ELISA kits (R&D, United States) according to the manufacturer’s instructions.

### Determination of Total Intestinal Mucosal Secretory Immunoglobulin A (SIgA)

The intestinal mucosa was gently scraped off with a glass slide. Mucosal tissue (0.5 g) was collected into an Eppendorf tube and 1 mL PBS (pH 7.4) containing 2 mg/mL protease inhibitor was added. Then, the mixture was sonicated for 20 s and centrifuged at 5000 rpm for 15 min. The supernatants were collected and concentrations of secretory immunoglobulin A (SIgA) were determined using the commercial ELISA kit (R&D, United States), following the manufacturer’s instruction (Bian et al., 2016).

### Statistical Analysis

The statistical significance of data comparisons was evaluated by one-way analysis of variance (ANOVA), followed by Duncan’s multiple range test or the least significant difference (LSD) test. Values of *P* < 0.05 were considered to be statistically significant.

## Results

### Survival Rate of Bifidobacteria Strains Under Simulated Digestive Tract Conditions

**FIGURE 1:**
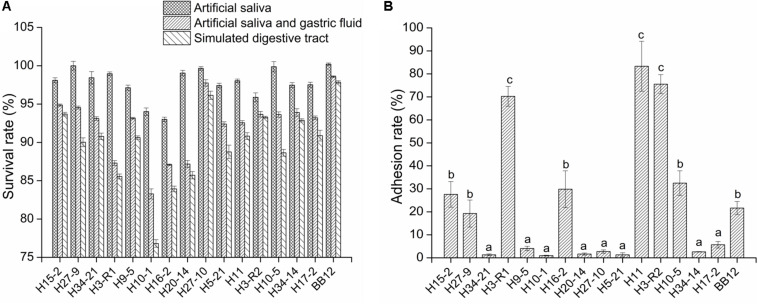
The survival rate of the bifidobacterial strains in simulated digestive tract conditions **(A)** and the adhesion ability of the bifidobacterial strains to intestinal epithelial cell **(B)** *in vitro*. All data are expressed as mean ± SD (*n* = 3 independent experiment). Different letters represent significant differences between different groups (*P* < 0.05).

Among the action of simulated saliva, gastric juice and intestinal juice, the artificial saliva treatment had less effect on the survival of bacteria than that of the other two digestive juices. Furthermore, the simulated gastric juice had the highest effect on bacteria of all three digestive juices. As compared to the reference strain BB12, all tested bifidobacteria strains exhibited a lower level of tolerance (*P* < 0.05), but the survival rates of the tested strains remained above 85% except for *B. longum* H10-1 and H16-2 after artificial intestinal fluid treatment (Figure 1A).

### Adhesion Ability Analysis of Bifidobacteria Strains to Caco-2

As shown in Figure 1B, the adhesion rates of six tested strains (H15-2, H3-R1, H16-2, H11, H3-R2, and H10-5) were above 20%. The adhesion rates of *B. longum* H3-R1 (70.26%), H11 (83.33%) and *B. bifidum* H3-R2 (75.25%) were significantly higher than other strains (*P* < 0.05), including reference strain BB12.

### Effect of Bifidobacteria Strains on the Proliferation of Lymphocyte and Phagocytosis of Macrophage *in vitro*

**FIGURE 2:**
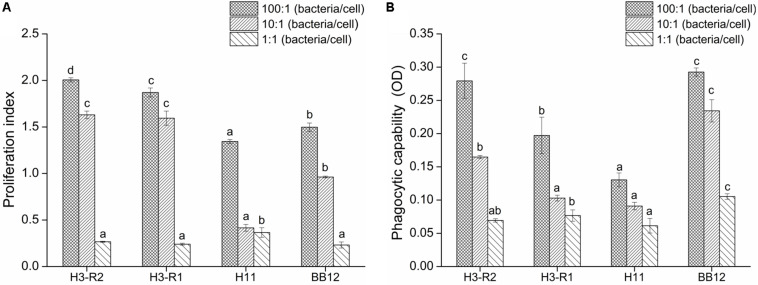
Effects of *B. bifidum* H3-R2 on proliferation of splenic lymphocyte **(A)** and phagocytosis of peritoneal macrophages **(B)** *in vitro*. All data are expressed as mean ± SD (*n* = 3 independent experiment). Different letters represent significant differences at the same ratio (bacteria/cells), *P* < 0.05.

*Bifidobacterium bifidum* H3-R2, *B. longum* H3-R1 and *B. longum* H11 were selected for *in vitro* immunization test. With the increase of populations of bacteria, the proliferation of lymphocyte and phagocytosis of macrophage were increased in each group. At the ratio of 10:1, the proliferation of *B. bifidum* H3-R2 and *B. longum* H3-R1 groups was significantly increased when compared with the BB12 group (*P* < 0.05), while there was no significant difference in the *B. bifidum* H3-R2 and *B. longum* H3-R1 groups. At the ratio of 100:1, the proliferation of *B. bifidum* H3-R2 group was significantly higher than other groups, including the reference strain BB12 group (*P* < 0.05) (Figure 2A). The phagocytosis of *B. bifidum* H3-R2 group was significantly higher than the *B. longum* H3-R1 and *B. longum* H11 groups at the ratio of 100:1 (*P* < 0.05), which was comparable to the reference strain BB12 group (Figure 2B).

### Effect of *B. bifidum* H3-R2 on the Body Weight, Thymus and Spleen Indexes

**FIGURE 3:**
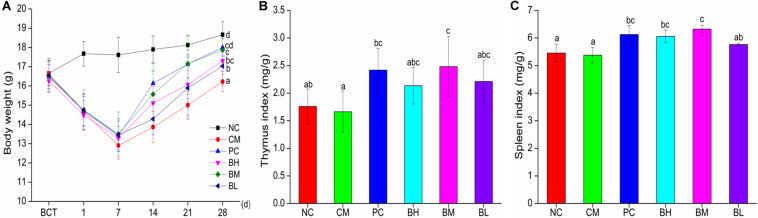
Effects of *Bifidobacterium bifidum* H3-R2 administration on body weight **(A)**, indexes of thymus **(B)**, and indexes of spleen **(C)** in mice. NC, normal control group; CM, CTX-induced model group; PC, positive control group; BH, high-dose group of *B. bifidum* H3-R2; BM, middle-dose group of *B. bifidum* H3-R2; BL, low-dose group of *B. bifidum* H3-R2. Typical data of one of the two independent animal experiments is shown (Supplementary Figures). All data are expressed as mean ± SD (six mice per group). Different letters represent significant differences (*P* < 0.05).

As shown in Figure 3A, after treatment with CTX for three consecutive days, the body weight of mice in the CTX-treated groups started to decrease and reached the lowest value on the 7th day. At the end of the experiments, compared with the CM group, the body weights of mice in the *B. bifidum*-treated groups showed better recovery, which was particularly obvious in the BM and PC groups (*P* < 0.05). Bifidobacteria-treated groups also improve in immune organ indexes (Figures 3B,C). The thymus indexes of BM were significantly increased when compared with the NC groups (*P* < 0.05), while there were no significant differences in the BM and PC groups. The spleen indexes of BH and BM groups were significantly higher than the NC groups (*P* < 0.05), and the spleen indexes of BH and BM groups were not significantly different from that of the PC group.

### Effect of *B. bifidum* H3-R2 on Histological Changes of Duodenum

**FIGURE 4:**
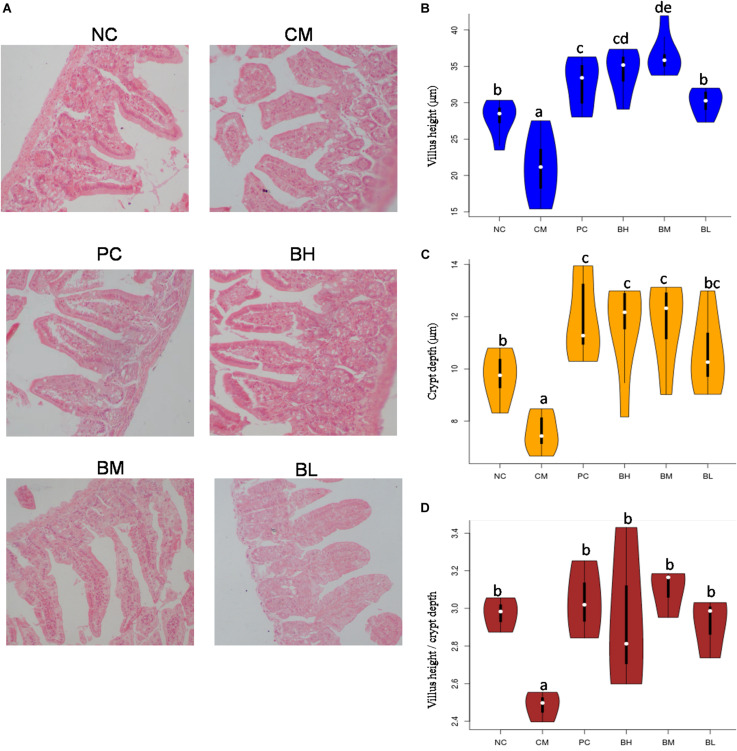
Effect of *Bifidobacterium bifidum* H3-R2 administration on intestinal histopathology **(A)**, height of intestinal villi **(B)**, depth of intestinal crypts **(C)** and ratios of villus height and crypt depth **(D)**. NC, normal control group; CM, CTX-induced model group; PC, positive control group; BH, high-dose group of *B. bifidum* H3-R2; BM, middle-dose group of *B. bifidum* H3-R2; BL, low-dose group of *B. bifidum* H3-R2. White dot represents median (50th percentile), black box represents interquartile range (25th and 75th percentiles). Mean values with unlike letters were significantly different (*P* < 0.05) (six mice per group).

The stained slices of the duodenum showed serious mucosa lesions in the CM group, including incomplete structure, loose arrangement, villus shortening and even shedding (Figure 4A). Compared with the CM group, all bifidobacteria-treated groups showed different degrees of improvement in the shape and structure of the intestine, with complete intestinal mucosa structure. Compared with the NC group, the length and depth of duodenal villi were significantly shorter and shallower in the CM group (*P* < 0.05), and the intervention of bifidobacteria groups were significantly higher than that in the CM group (*P* < 0.05) (Figures 4B,C). Moreover, the length of villi in the BM group was significantly higher than that in the NC and PC groups (*P* < 0.05). The ratio of villi and crypts in the CM group were significantly lower than that in the NC group, while all the *B. bifidum*-treated groups were not significantly different from the NC and PC groups (Figure 4D).

### Effect of *B. bifidum* H3-R2 on Splenocyte Proliferation and NK Cell Activity

**FIGURE 5:**
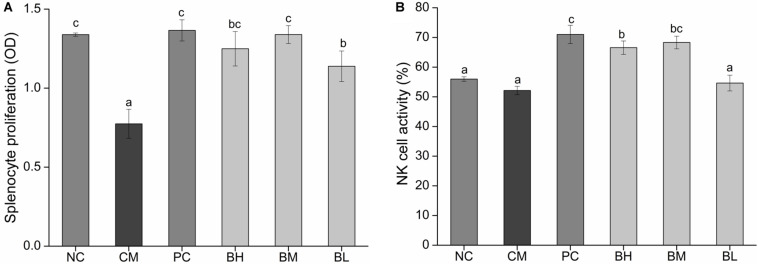
Effect of *Bifidobacterium bifidum* H3-R2 administration on proliferation level of splenic lymphocyte **(A)** and the activity of NK cell **(B)** in mice. NC, normal control group; CM, CTX-induced model group; PC, positive control group; BH, high-dose group of *B. bifidum* H3-R2; BM, middle-dose group of *B. bifidum* H3-R2; BL, low-dose group of *B. bifidum* H3-R2. Data are expressed as mean ± SD (six mice per group). Different letters indicate that there is a significant difference (*P* < 0.05).

The proliferation of splenic lymphocytes in the CM group was significantly lower than the NC group. However, the proliferation of splenic lymphocytes was significantly enhanced in the bifidobacteria-treated groups when compared with that in the CM group (*P* < 0.05) (Figure 5A). In particular, the BM and BH groups showed no significant difference when compared with the NC and PC groups. As shown in Figure 5B, the NK cell activity of the CM group was not significantly different from the NC group, however, the NK cell activity of BH and BM groups were significantly enhanced compared with the CM group (*P* < 0.05). The BM group was comparable to the PC group in NK cell activity (*P* > 0.05).

### Effect of *B. bifidum* H3-R2 on Macrophage Viability

**FIGURE 6:**
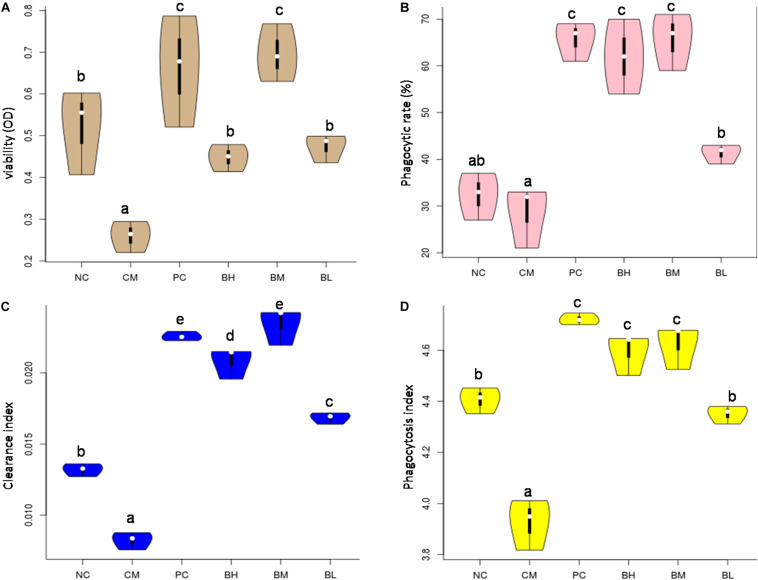
Effects of *Bifidobacterium bifidum* H3-R2 on macrophages *in vivo*. The viability of macrophage **(A)**, phagocytic ability of macrophage **(B)**, clearance index **(C)**, and phagocytosis index **(D)**. NC, normal control group; CM, CTX-induced model group; PC, positive control group; BH, high-dose group of *B. bifidum* H3-R2; BM, middle-dose group of *B. bifidum* H3-R2; BL, low-dose group of *B. bifidum* H3-R2. White dot represents median (50th percentile), black box represents interquartile range (25th and 75th percentiles). Mean values with unlike letters were significantly different (*P* < 0.05) (five mice per group).

The viability of the CM group was significantly decreased when compared with the NC group (*P* < 0.01) (Figure 6A). The viability of the bifidobacteria-treated groups was significantly higher than that of the CM group (*P* < 0.05). Among them, there was no significant difference between BM group and PC group, but they were significantly higher than other groups (*P* < 0.05).

### Effect of *B. bifidum* H3-R2 on Phagocytic Activity of Macrophages

The phagocytic ability of macrophages against CRBCs, the rates of carbon clearance and phagocytosis index were used as the indexes of phagocytosis to evaluate the effect of bifidobacteria on phagocytic activity in immunosuppressed mice. The phagocytic ability of macrophages treated with different doses of *B. bifidum* H3-R2 was significantly increased when compared with the CM group (*P* < 0.05) (Figure 6B). In particular, the phagocytic ability of macrophages in the BM and BH groups were approximately twice as high as the NC group (*P* < 0.05), being equivalent to the PC group. The clearance index and phagocytosis index of the CM group were significantly lower than the NC group, and the indexes were significantly higher in the bifidobacteria-treated groups than in the CM and NC groups (*P* < 0.05) (Figures 6C,D). Furthermore, the clearance index and phagocytosis index of the BM group were comparable to the PC group.

### Effect of *B. bifidum* H3-R2 on Production of Cytokines

**FIGURE 7:**
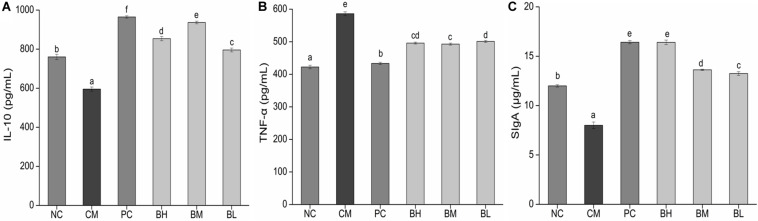
Effects of *Bifidobacterium bifidum* H3-R2 administration on the secretion of IL-10 **(A)**, TNF-α **(B)**, and SIgA **(C)** in mice. NC, normal control group; CM, CTX-induced model group; PC, positive control group; BH, high-dose group of *B. bifidum* H3-R2; BM, middle-dose group of *B. bifidum* H3-R2; BL, low-dose group of *B. bifidum* H3-R2. Values are expressed in mean ± SD (six mice per group). The different letters represent significant differences (*P* < 0.05).

The concentration of IL-10 in the CM group was significantly lower than in the NC group, while the concentration of TNF-α was significantly increased (*P* < 0.05) (Figures 7A,B). The concentration of IL-10 in the bifidobacteria-treated groups was significantly increased compared with the CM and NC groups (*P* < 0.05), while the concentration of TNF-α was significantly lower than that in the CM group (*P* < 0.05).

### Effect of *B. bifidum* H3-R2 on Total Intestinal Mucosal SIgA

The concentration of intestinal mucosal SIgA in the CM group was significantly lower than that in the NC group (*P* < 0.05) (Figure 7C), which might be due to the destruction of mucosal tissue by CTX. The concentration of SIgA in all the bifidobacteria-treated groups were significantly higher than that in the NC groups (*P* < 0.05). The BH group was not significantly different from the PC group and was significantly higher than other groups (*P* < 0.05).

## Discussion

Commercial strain BB12, confirmed to have strong acid and bile resistance, excellent intestinal adhesion, enhanced barrier function and immune interaction (Mohan et al., 2006; Guo et al., 2009; Holscher et al., 2012; Rizzardini et al., 2012), was selected as PC. It is well known that beneficial effects of probiotics are attainable if bacteria are able to survive in the adverse conditions of the digestive tract (Turchi et al., 2013; Rabah et al., 2017), therefore, we screened potential probiotics *in vitro* by determining survival rate in simulated digestive tract conditions. The survival rates of the tested strains were above 85% except for *B. longum* H10-1 and H16-2, implying that these strains with positive tolerance of the digestive tract may be advantageous for survival in the host gut. Adhesion of probiotic bacteria to the intestinal epithelium is an important characteristic as it promotes colonization and immunomodulatory effects (Wang et al., 2013; Monteagudo-Mera et al., 2019). *B. bifidum* H3-R2, *B. longum* H3-R1 and *B. longum* H11 had better adhesive properties than the reference strain BB12. This finding implied that these three bifidobacteria strains could effectively adhere to intestinal epithelial cells. The components of bifidobacteria act as adhesive factors, including lipoteichoic acids, carbohydrates, extracellular proteins, and surface proteins located on the outer surface of the cell wall (Fanning et al., 2012a; González-Rodríguez et al., 2012; Alp and Kuleaşan, 2019).

Splenic lymphocytes and macrophages are a crucial part of specific immunity and non-specific immunity in the immune system (Larry et al., 2017). The activity and proliferation of splenic lymphocytes are often used as parameters for assessing cell immune function. In this study, the proliferation of lymphocyte was positively correlated with the amount of bifidobacteria *in vitro*. The proliferation of lymphocyte of the *B. bifidum* H3-R2 group was significantly higher than other groups at the ratio of 100:1, indicating that *B. bifidum* H3-R2 may have a great effect on the immune response. The phagocytosis of Macrophages is a crucial defense mechanism, which protects against pathogen invasions (Miyata and Eeden, 2011; Liu et al., 2016). The *B. bifidum* H3-R2 had an obvious promoting effect on the phagocytosis of macrophages when compared with the *B. longum* H3-R1 and *B. longum* H11 at the ratio of 100:1, which was comparable to the reference strain BB12. The results suggested that *B. bifidum* H3-R2 could play an important role in immune defense. Phagocytic activity of macrophages was enhanced after co-culture with lactic acid bacteria, which was consistent with the previous study (Rocha-Ramírez et al., 2017). One hypothetical pathway for how probiotics modulate immune function in the intestine is that M (microfold) cells can transport probiotics by transcytosis. Macrophages are present immediately below M cells to engulf probiotics and then trigger immune responses (Shida and Nanno, 2008).

*Bifidobacterium bifidum* H3-R2 had the positive potential to regulate immune cell activity *in vitro* and was selected to evaluate its immunomodulatory effects on immunosuppressed mice. After treatment with CTX for 3 days, the mice in CTX-treated groups showed some clinical symptoms, such as moist feces, reduced food intake, hair loss and lethargy. The mice showed a gradual return to normal when treated with different doses of bifidobacteria.

Thymus and spleen are important immune organs in the body. The thymus is the location of proliferation, differentiation and maturation of T cells, and the spleen is the site where T and B cells receive antigen stimulation to stimulate the immune response. The immune organs indexes are often used as preliminary indicator to evaluate the body’s immune function (Sun et al., 2016; Han et al., 2019). In this study, the spleen and thymus indexes of the CM group mice were comparable to the NC group, implying that CTX may result in decline in organ function to a certain extent and the body may possess a self-healing ability because of autoreactive cells (L’Age-Stehr and Diamanstein, 1978). However, the immune organs indexes of the BM or BH group were significantly higher than that of the NC group, indicating that *B. bifidum* H3-R2 can promote the development of immune organs and enhance the host immune system. It has been confirmed that some probiotics significantly elevate the immune organs index and enhance their development (Nakamura et al., 2012; Ren et al., 2015). CTX can destroy immune cells and thus affect mucosal immunity (Ding et al., 2019), however, *B. bifidum* H3-R2 can alleviate and repair the damage, thus playing a role in protecting the intestinal mucosal tissue.

The proliferation of splenic lymphocytes was inhibited in the CM group, which was enhanced to normal level by the oral administration of *B. bifidum* H3-R2, except for in the BL group, implying that middle and high doses of *B. bifidum* H3-R2 could restore the activity of splenic lymphocytes and enhanced organ immune function. The NK cells are involved in the immune response to destroy tumor cells and virus-infected cells directly without tumor-specific antigen recognition (Bowen et al., 2018). The middle and high doses of *B. bifidum* H3-R2 could enhance the NK cell activity at normal level, but low dose of *B. bifidum* H3-R2 showed no effect. The viability and phagocytosis index of the CM group were decreased when compared with the NC group, indicating that CTX could inhibit the activity of macrophages and affect host non-specific immunity. The immune response requires a concomitant and coordinated regulation of the energetic metabolism. Macrophage activity affects energy metabolism, and the fast generation of adenosine triphosphate (ATP) through glycolysis in order for the macrophages to cope with highly proliferative bacterial infections (Verdeguer and Aouadi, 2017). Oral administration of *B. bifidum* H3-R2 could restore the activity of macrophages in the immunosuppressed host and thus participate in the immune response, and the BM group was comparable to the PC group in the activity of macrophages. Different doses of *B. bifidum* H3-R2 enhanced the function of the immune cells in immunosuppressed mice, which demonstrated that it had great effect on immune regulation. *B. bifidum* H3-R2 might improve the ability of host immune defense by promoting the splenic lymphocyte proliferation and the activity of phagocytes and NK cells. Similarly, it was reported that the activity of phagocytes was significantly enhanced after the volunteers were given BB12 in the human trial (Schiffrin et al., 1997). Furthermore, *Lactobacillus rhamnosus* HN001 and *Lactobacillus acidophilus* NCFM could significantly enhance NK cell activity in healthy elderly, while *B. longum* BB536 had no significant effect (Ibrahim et al., 2010; Akatsu et al., 2013). Therefore, not all strains have the ability to stimulate the activity of immune cells and the characteristics may reflect the strain-specific immune regulation. Specific bifidobacteria had an obvious effect on stimulating the activity of immune cells with the active substances such as protein components, peptidoglycan, lipoteichoic acid and exopolysaccharides (Fanning et al., 2012b; Hidalgo-Cantabrana et al., 2017; Ruiz et al., 2017).

Cytokines, secreted by stimulating immune cells and certain non-immune cells, are involved in the immune response (Belkaid and Harrison, 2017). IL-10 exerts a strong suppressive effect on antigen-presenting cells, Th1 lymphocytes and the production of inflammatory mediators (Feng et al., 2002; Medina et al., 2008). TNF-α and IL-10 are two pleiotropic cytokines which are mutually regulated and play opposite roles in inflammatory responses, and therefore their relative balance is relevant for controlling immune deviation (López et al., 2010). In this study, CTX has a negative influence on immune function, decreasing the capacity of immune cells to increase secretion of pro-inflammatory cytokine TNF-α. Each dose of *B. bifidum* H3-R2 was able to promote the production of anti-inflammatory cytokine IL-10 and inhibit the secretion of pro-inflammatory cytokine TNF-α, suggesting that *B. bifidum* H3-R2 could regulate cytokines to reduce inflammation. *In vitro*, exposure of dendritic cells to *B. bifidum* LMG 13195 or membrane vesicles from this strain of bacteria promotes the differentiation of naive T cells and triggers production of IL-10. This effect might contribute to local immunosuppressive effects in the gut (López et al., 2012; Routy et al., 2018). The ability of probiotic bacteria to downregulate TNF-α production and upregulate IL-10 has been reported previously. Previous results have shown that probiotic bacteria could downregulate TNF-α production and upregulate IL-10 in a rat model of trinitrobenzene sulfonic acid (TNBS)-induced colitis (Peran et al., 2005). Thus, bifidobacteria could contribute to immunity by regulating inflammatory cytokines.

Secretory immunoglobulin A is the major immune effector in the intestinal mucus layer, which protects the body by preventing microorganisms or their toxic products from attaching to the surface epithelium (Brandtzaeg, 2009). SIgA is a chimeric molecule generated by the combined activity of both plasma cells and polymeric Ig receptor (pIgR)-expressing (mostly epithelial) cells. Therefore, production and secretion of SIgA is not only determined by rates of antibody production by plasma cells but additionally influenced by pIgR expression and activity (Pabst and Slack, 2020). Mucosal damage may cause the decrease of SIgA levels observed in this study, and a deficiency of SIgA in the intestinal mucosa depresses the immune response against infection. The villus length and crypt depth of the intestine directly reflect mucosal barrier development, and their ratio indirectly affects the absorption and utilization of substances (Perez-Lopez et al., 2016; Xie et al., 2016). Our results showed that each dosage group could enhance SIgA above normal level. The BM and BH groups were better than the BL group in the terms of restored villus length and crypt depth. *B. bifidum* H3-R2 alleviated the mucosal damage caused by CTX in immunosuppressed mice, increased the villus length and crypt depth to improve mucosa damage and promoted production of SIgA, thus *B. bifidum* H3-R2 had a protective effect on mucosal immunity. Yang et al. (2009) reported that *Bifidobacterium adolescentis* BBMN23 and *B. longum* BBMN68 could significantly increase the villus length and crypt depth and the number of mucosal immunity-associated cells. In addition, oral administration in rats of bifidobacteria after thermal injury resulted in improved mucosal injury and increased the levels of SIgA in the small intestine, which were associated with improved gut barrier and decreased bacterial translocation (Wang et al., 2006).

In this study, the adhesion ability of *B. bifidum* H3-R2 was significantly higher than that of BB12, but the immunoregulatory capability of *B. bifidum* H3-R2 was comparable to BB12 in immunosuppressed mice, it was inferred that adhesion-related proteins may not be main substances to stimulate the cellular immune activity. In addition, the immune effect of high doses of *B. bifidum* H3-R2 was similar to that of medium doses. This may be that mice have limited tolerance of strains. It is certain that *B. bifidum* H3-R2 has a positive immune regulatory effect on immunosuppressed mice.

## Conclusion

In this study, we obtained a potential probiotic *B. bifidum* H3-R2. *In vitro*, it showed a positive tolerance to digestive tract conditions, adhesion ability to intestinal epithelial cells and a regulatory effect on immune cell activity. *In vivo*, the administration of *B. bifidum* H3-R2 could restore body weight, improve immune cell activity, balance expression of inflammatory cytokines and enhance the production of SIgA. The present results indicated that *B. bifidum* H3-R2 had promising immune-protective effects *in vitro* and *in vivo*, which could provide basic information and suggested direction for future applied research. *B. bifidum* H3-R2 holds an important potential to trigger immunomodulatory responses involved in the maintenance of our healthy physiological state. However, these responses need more research to elucidate the mechanism of molecular communication between *B. bifidum* H3-R2 and host immune system.

## Data Availability Statement

All datasets generated for this study are included in the article/Supplementary Material.

## Ethics Statement

The animal study was reviewed and approved by the Institutional Animal Care and Use Committee of the Northeast Agricultural University.

## Author Contributions

XM and BL designed and conducted the study. JS and FW analyzed the data and wrote the manuscript. LZ prepared the Figures. All authors contributed to the article and approved the submitted version.

## Conflict of Interest

The authors declare that the research was conducted in the absence of any commercial or financial relationships that could be construed as a potential conflict of interest.
