# Supplementary material for: Potential Immunomodulatory Activity of a Selected Strain Bifidobacterium bifidum H3-R2 as Evidenced in vitro and in Immunosuppressed Mice
Source: Front Microbiol. 2020 Sep 1;11:2089. doi: 10.3389/fmicb.2020.02089 (PMC7491056; doi:10.3389/fmicb.2020.02089)
Supplement: Supplementary file 1 [file Data_Sheet_1.PDF]

Supplementary Figures

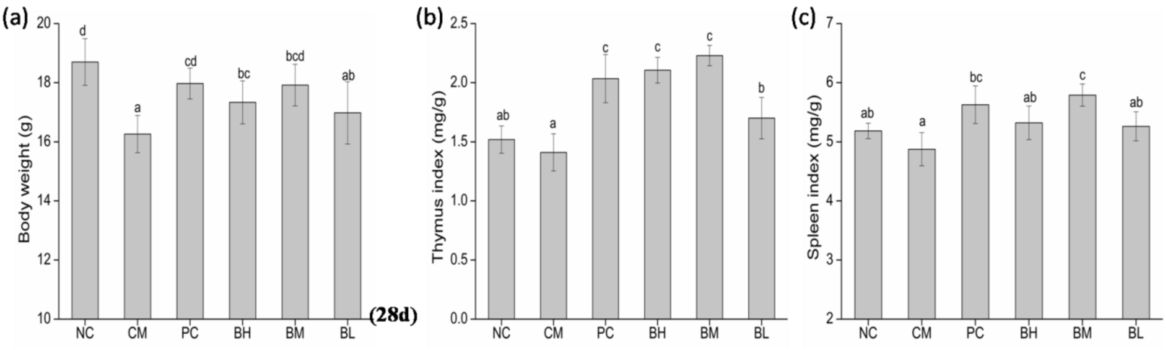

Supplementary Figure 1 Effects of *B. bifidum* H3-R2 administration on the body weight (a), indexes of thymus (b) and indexes of spleen (c) NC: normal control group; CM: CTX-induced model group; PC: positive control group; BH: high-dose group of *B. bifidum* H3-R2; BM: middle-dose group of *B. bifidum* H3-R2; BL: low-dose group of *B. bifidum* H3-R2. All data are expressed as mean ± SD (6 mice per group). Different letters represent significant differences (P < 0.05).

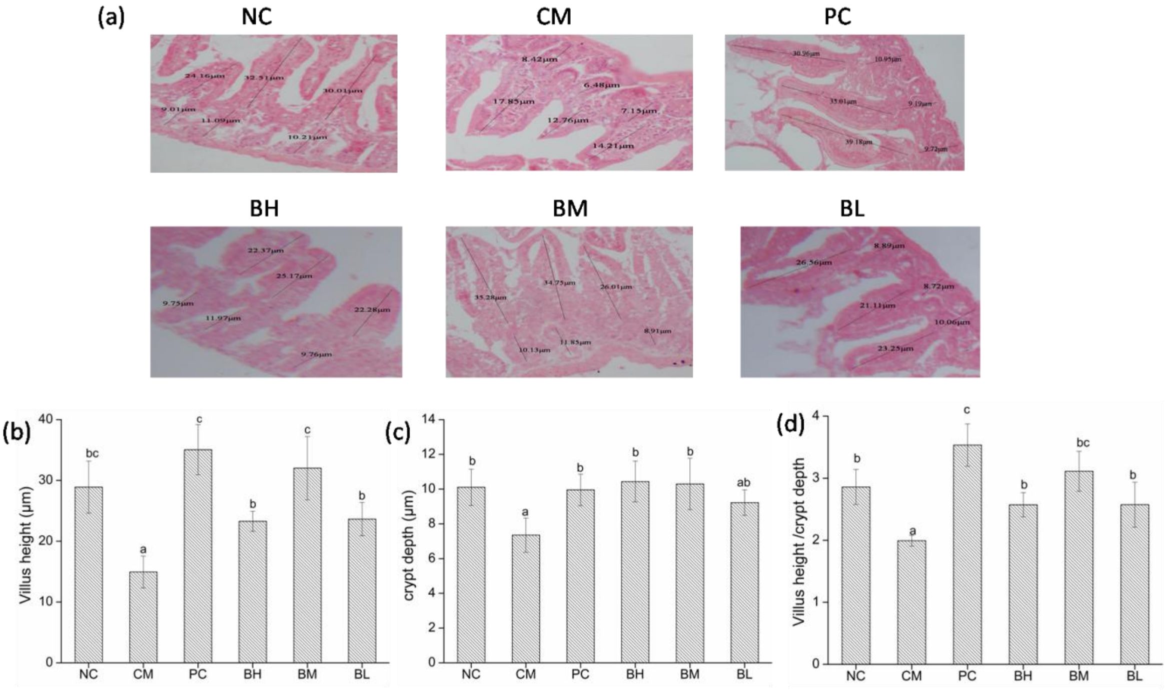

Supplementary Figure 2 Effect of *B. bifidum* H3-R2 administration on intestinal histopathology (a), height of villi (b), depth of crypts (c) and ratios of villi and crypts (d). NC: normal control group; CM: CTX-induced model group; PC: positive control group; BH: high-dose group of *B. bifidum* H3-R2; BM: middle-dose group of *B. bifidum* H3-R2; BL: low-dose group of *B. bifidum* H3-R2. Values are shown as mean ± SD (6 mice per group). Different letters represent significant differences, P < 0.05.

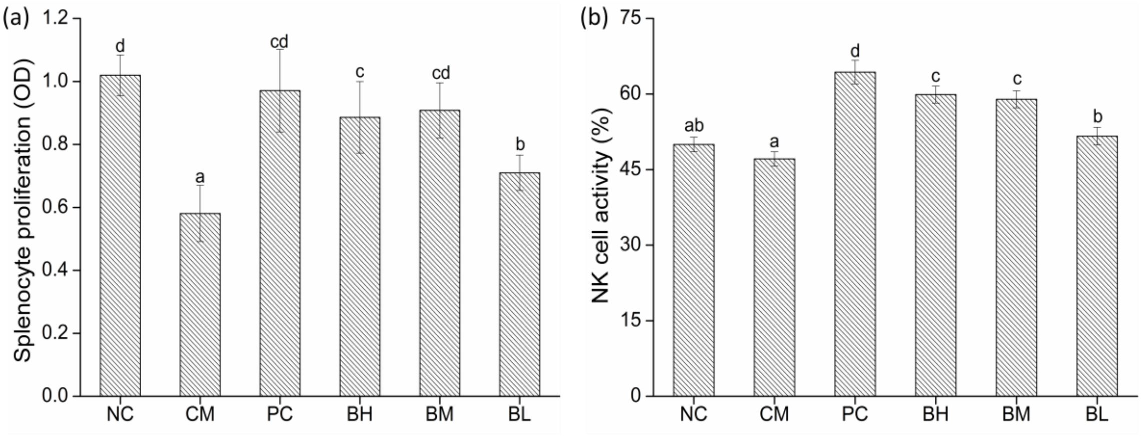

Supplementary Figure 3 Effect of *B. bifidum* H3-R2 administration on proliferation level of splenic lymphocyte (a) and the activity of NK cell (b) in mice. NC: normal control group; CM: CTX-induced model group; PC: positive control group; BH: high-dose group of *B. bifidum* H3-R2; BM: middle-dose group of *B. bifidum* H3-R2; BL: low-dose group of *B. bifidum* H3-R2. Data are expressed as mean ± SD (6 mice per group). Different letters indicate that there is a significant difference ($P < 0.05$).

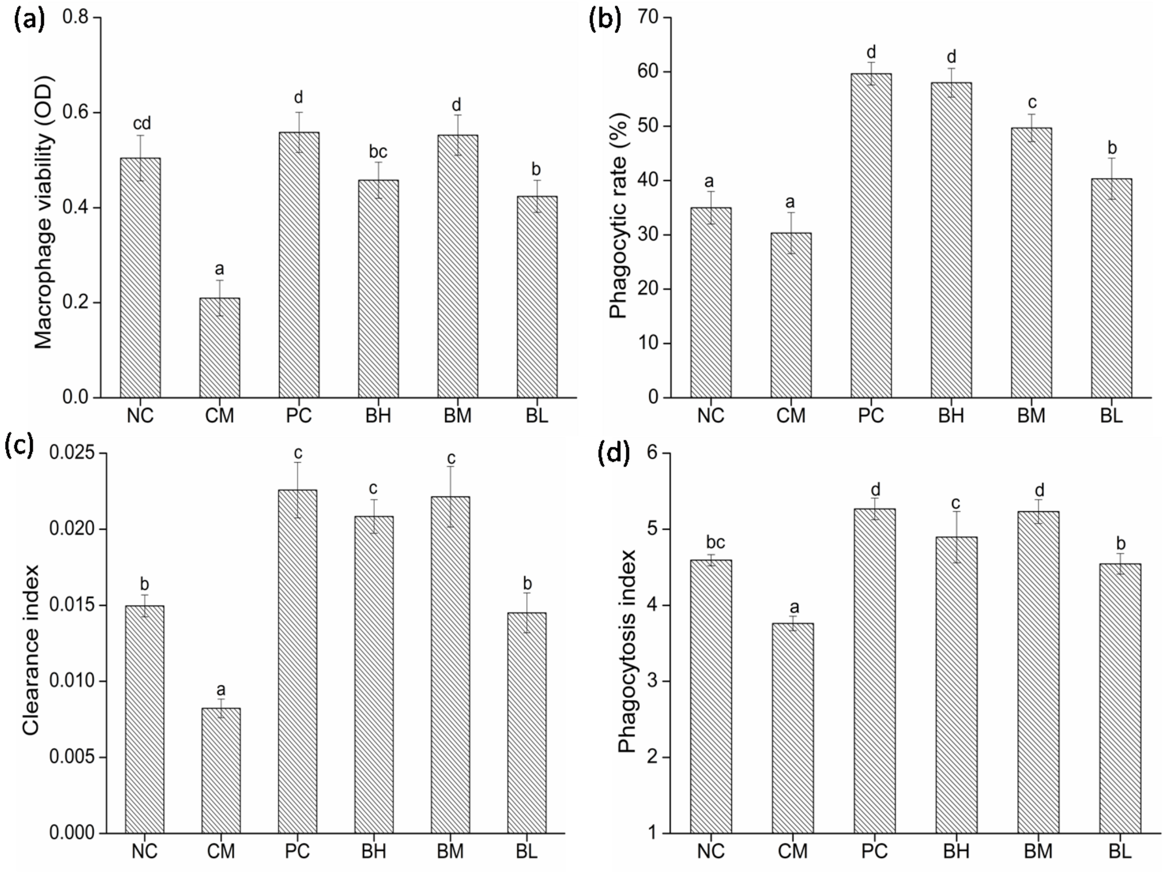

Supplementary Figure 4 Effects of *B. bifidum* H3-R2 on macrophages in mice. The viability of macrophage (a), phagocytic ability of macrophage (b), clearance index (c) and phagocytosis index (d). NC: normal control group; CM: CTX-induced model group; PC: positive control group; BH: high-dose group of *B. bifidum* H3-R2; BM: middle-dose group

of *B. bifidum* H3-R2; BL: low-dose group of *B. bifidum* H3-R2. Data are expressed as mean ± SD (6 mice per group). Different letters indicate that there is a significant difference (P < 0.05).

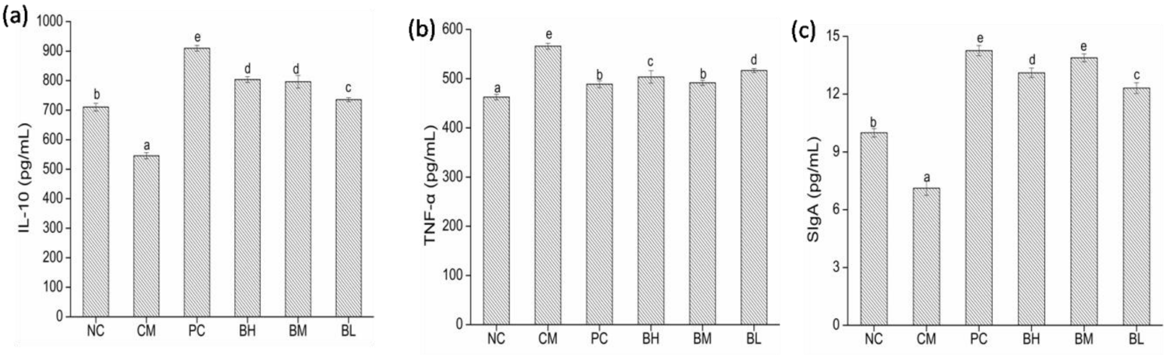

Supplementary Figure 5 Effects of *B. bifidum* H3-R2 administration on the secretion of IL-10 (a), TNF-α (b) and SIgA (c) in mice. NC: normal control group; CM: CTX-induced model group; PC: positive control group; BH: high-dose group of *B. bifidum* H3-R2; BM: middle-dose group of *B. bifidum* H3-R2; BL: low-dose group of *B. bifidum* H3-R2. Values are expressed in mean ± SD (6 mice per group). The different letters represent significant differences (P < 0.05).
